# Identification of plicamycin, TG02, panobinostat, lestaurtinib, and GDC-0084 as promising compounds for the treatment of central nervous system infections caused by the free-living amebae *Naegleria*, *Acanthamoeba* and *Balamuthia*

**DOI:** 10.1016/j.ijpddr.2019.10.003

**Published:** 2019-10-22

**Authors:** Monica M. Kangussu-Marcolino, Gretchen M. Ehrenkaufer, Emily Chen, Anjan Debnath, Upinder Singh

**Affiliations:** a Division of Infectious Diseases, Department of Internal Medicine, Stanford University, Grant Building, S-143, 300 Pasteur Drive, Stanford, CA, 94305, USA; b uHTS Laboratory Rm 101, 11119 N Torrey Pines Rd. Calibr, A Division of the Scripps Research Institute, La Jolla, CA, 92037, USA; c Center for Discovery and Innovation in Parasitic Diseases, Skaggs School of Pharmacy and Pharmaceutical Sciences, University of California, San Diego, La Jolla, CA, 92093, USA; d Department of Microbiology and Immunology, Stanford University, Stanford, CA, 94305, USA

**Keywords:** *Naegleria*, *Acanthamoeba*, *Balamuthia*, ReFRAME library, CNS infection, Drug screening

## Abstract

The free-living amebae *Naegleria*, *Acanthamoeba*, and *Balamuthia* cause rare but life-threatening infections. All three parasites can cause meningoencephalitis. *Acanthamoeba* can also cause chronic keratitis and both *Balamuthia* and *Acanthamoeba* can cause skin and systemic infections. There are minimal drug development pipelines for these pathogens despite a lack of available treatment regimens and high fatality rates. To identify anti-amebic drugs, we screened 159 compounds from a high-value repurposed library against trophozoites of the three amebae. Our efforts identified 38 compounds with activity against at least one ameba. Multiple drugs that bind the ATP-binding pocket of mTOR and PI3K are active, highlighting these compounds as important inhibitors of these parasites. Importantly, 24 active compounds have progressed at least to phase II clinical studies and overall 15 compounds were active against all three amebae. Based on central nervous system (CNS) penetration or exceptional potency against one amebic species, we identified sixteen priority compounds for the treatment of meningoencephalitis caused by these pathogens. The top five compounds are (i) plicamycin, active against all three free-living amebae and previously U.S. Food and Drug Administration (FDA) approved, (ii) TG02, active against all three amebae, (iii and iv) FDA-approved panobinostat and FDA orphan drug lestaurtinib, both highly potent against *Naegleria*, and (v) GDC-0084, a CNS penetrant mTOR inhibitor, active against at least two of the three amebae. These results set the stage for further investigation of these clinically advanced compounds for treatment of infections caused by the free-living amebae, including treatment of the highly fatal meningoencephalitis.

## Highlights

• Focused screen of ReFRAME library (159 compounds) on three free-living amebae.
• 16 priority compounds selected based on potency, advanced clinical studies and CNS penetration.
• Identified compounds with fast killing kinetics against *Naegleria* and *Balamuthia*.
• Identified compounds capable of delaying *Balamuthia* trophozoite recrudescence.
• 5 compounds of high interest for treatment of CNS infections with free-living amebae.

## Graphical abstract

**Figure:**
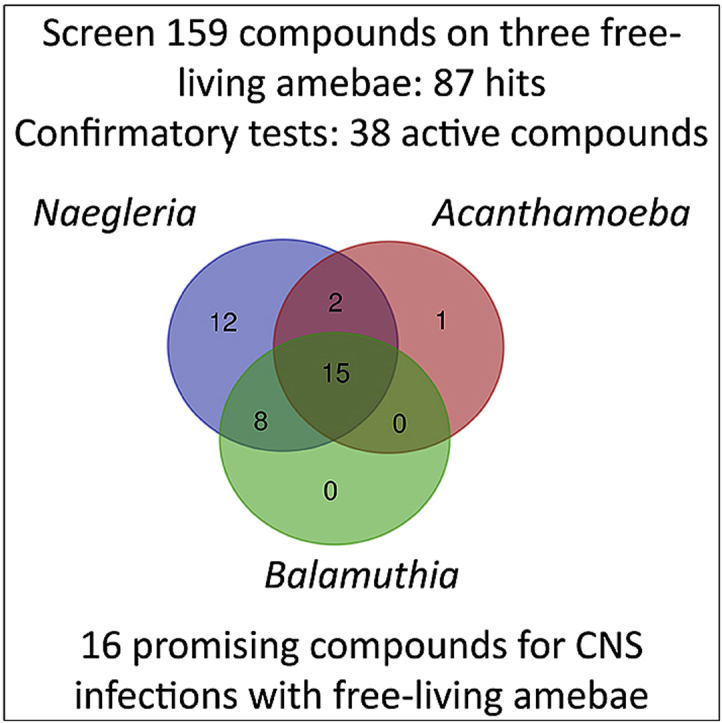

## 1 Introduction

*Naegleria fowleri*, *Acanthamoeba* spp. and *Balamuthia mandrillaris* are free-living amebae widely spread in the environment that can act as opportunistic pathogens and cause deadly human infections. However, despite their highly pathogenic nature, there are no optimal established clinical therapies. Infection with *N. fowleri* results in a rapidly progressive Primary Amebic Meningoencephalitis (PAM), which is a severe brain infection and can lead to death in 5–7 days (Capewell et al., 2015). *Acanthamoeba* spp. and *B. mandrillaris* cause a more indolent process, Granulomatous Amebic Encephalitis (GAE), which is more common in immunosuppressed individuals, although a few cases of *Balamuthia* infection in immunocompetent patients have been reported (Jung et al., 2004; Visvesvara et al., 2007). These two parasites can also disseminate to the skin, lungs and other organs (Khan, 2006). *Acanthamoeba* spp. can also cause amebic keratitis, a severe, unrelenting disease among contact lens wearers, that often necessitates corneal transplantation and can result in blindness (Awwad et al., 2007). Despite the severity of the diseases caused by these amebae, these organisms are understudied and have extremely poor treatment options (Visvesvara, 2010).

Young children are the most affected by *Naegleria* PAM, commonly through recreational fresh water exposure (Yoder et al., 2010). *N. fowleri* accesses the central nervous system (CNS) via the olfactory nerves after entering the nostrils through contaminated water. In the United States from 1962 to 2018, 145 cases were documented with only four survivors, a fatality rate of over 97% (Capewell et al., 2015). The high mortality is a consequence of delayed diagnosis, lack of efficacious treatment and difficulty of delivering drugs to the CNS. Current treatment protocols rely on combination therapy, including miltefosine, amphotericin B, azithromycin, fluconazole, rifampin but the regimens are not uniformly successful and it is clear that improved regimens are still needed (CDC, 2013b; Cope et al., 2016; Linam et al., 2015).

Both *Acanthamoeba* spp. and *B. mandrillaris* can enter either via respiratory tract or broken skin and invade the CNS and cause GAE (CDC, 2017; Siddiqui and Khan, 2015). Current GAE treatment consists of experimental multi-drug regimen based on a few previous successful cases. For GAE caused by *Acanthamoeba*, the CDC recommends pentamidine, sulfadiazine, flucytosine, and either fluconazole or itraconazole, while for *Balamuthia* infection, the recommended treatment is flucytosine, pentamidine, fluconazole and sulfadiazine plus either azithromycin or clarithromycin (CDC, 2013a). Miltefosine has also recently been used for treatment of GAE with *Acanthamoeba* or *Balamuthia* (CDC, 2017). Treatment for *Acanthamoeba* keratitis has some efficacy against the replicative trophozoite stage and include topical polyhexamethylene biguanide (PHMB) or chlorhexidine along with a diamidine (either propamidine or hexamidine) (Visvesvara, 2010). However, the presence of *Acanthamoeba* cysts, resistant to antimicrobials, can lead to the recurrence of keratitis. No current drug is effective against both the trophozoite and cyst stages of *Acanthamoeba*.

In addition to these therapeutic challenges, it can also be difficult to diagnose PAM and GAE. High physician vigilance and awareness of the epidemiologic factors are essential. In addition, tissue samples (CSF or brain biopsy for PAM and GAE), skin biopsies (for disseminated *Acanthamoeba* or *Balamuthia* infection), or corneal scrapings (for *Acanthamoeba* keratitis) are often needed (Awwad et al., 2007). Specialized diagnostic tests for parasite identification done at the CDC can also be helpful (CDC, 2017). Considering the diagnostic and therapeutic challenges, identification of improved regimens as well as compounds that work against all three free-living amebae would be an important advance.

Given their rarity, there is a lack of interest by the pharmaceutical industry in developing drugs against free-living amebic infections. The traditional route of drug development (target identification, validation, drug formulation, clinical studies) is a long and expensive road with the average cost per drug brought to the market of ~$2.6 billion (DiMasi et al., 2016). Screening of previously developed small molecules, which have established toxicology data, or which have been in clinical trials for other indications, can reduce development costs by speeding time to clinical use. Similar approaches have identified a new potential compound auranofin, an FDA approved drug with activity against multiple protozoan parasites including *Entamoeba histolytica*, *Giardia lamblia, Cryptosporidium parvum, Trichomonas vaginalis*, *Toxoplasma gondii* and *Leishmania donovani* (Andrade et al., 2014; Debnath et al., 2012a, 2013; Hopper et al., 2016; Ilari et al., 2012; Tejman-Yarden et al., 2013), indicating that success with a similar algorithm against the free-living amebae may be successful.

In this work we screened a subset of compounds available in the Repurposing, Focused Rescue and Accelerated MEdicinal chemistry (ReFRAME) library, as well as a few additional related compounds, against trophozoites of the three free-living amebae *N. fowleri*, *A. castellanii* and *B. mandrillaris*. This library was previously demonstrated to be composed of high-value compounds for accelerated drug development efforts and has been successfully used to identify new drug options for *Cryptosporidium parvum* (Janes et al., 2018). We previously performed a primary screen of the ReFRAME library against a parasitic ameba *Entamoeba histolytica* and the active compounds identified from this screen (reframedb.org) (Kangussu-Marcolino et al., in preparation) were selected for this study to test their activity against three free-living amebae. Following confirmatory tests, we prioritized 16 compounds that possess CNS permeability or showed high potency against these CNS-invasive pathogens. We also evaluated the killing kinetics of selected compounds and performed a recrudescence study to determine if a brain-infecting parasite can recover after the treatment of compounds. We identified five high-value compounds, plicamycin, TG02, panobinostat, lestaurtinib, and GDC-0084, based on advanced clinical stages, CNS permeability, ability to kill *Naegleria* and *Balamuthia* rapidly, and prevented recovery of *Balamuthia* following drug treatment. Given that many of these compounds have significant clinical use and known brain penetration, this effort is a significant advance for the treatment of these neglected but fatal human infections.

## 2 Materials and methods

### 2.1 Parasite culture

*Naegleria* *fowleri*:

Trophozoites of pathogenic *N. fowleri* strain KUL (ATCC) were axenically cultured in Nelson's medium supplemented with 10% FBS at 37 °C (Debnath et al., 2017, 2018). Trophozoites were counted using a hemocytometer. All the experiments were performed using trophozoites harvested during the logarithmic phase of growth.

*Acanthamoeba castellanii*: *A. castellanii* strain Ma trophozoites belonging to T4 genotype (ATCC) were cultured at 28 °C in PYG medium according to a modified technique (Debnath et al., 2014). Trophozoites were harvested at 48 h when cells were in logarithmic phase of growth. Since T4 is the predominant genotype in brain infections caused by *Acanthamoeba* (Booton et al., 2005), we selected a strain representing T4 genotype.

*Balamuthia mandrillaris*: Trophozoites (ATCC strain PRA-291, gift from Joseph L. DeRisi lab) were grown in axenic Cerva's medium (Lares-Jimenez et al., 2015) at 37 °C and 5% CO_2_ and sub-cultured every 3–4 days. All the experiments were performed using trophozoites harvested during the logarithmic phase of growth.

HFF-1 cells (gift from John Boothroyd lab) were cultured in DMEM with 1  g/L glucose, Sodium Pyruvate and L-glutamine (Gibco) completed with 10% (vol/vol) FBS, and 100 U/mL penicillin/streptomycin at 37 °C with 5% CO_2_.

### 2.2 Susceptibility of amebae and drug testing

*Naegleria* and *Acanthamoeba*: The compounds were screened against *N. fowleri* and *A. castellanii* using established parameters (Debnath et al., 2017). For the initial 8-point dose response study, 50 μL of *N. fowleri* (5000 trophozoites) and *A. castellanii* (2500 trophozoites) were added to 96-well plated compounds to yield final concentrations spanning from 0.078 μM to 10 μM. Negative controls in the screen plates contained 0.5% DMSO and positive controls contained 50 μM of amphotericin B (Amphotericin B solubilized, Sigma-Aldrich) for *N. fowleri* and 50 μM of chlorhexidine for *A. castellanii*. Assay plates were incubated for 48 h at 37 °C for *N. fowleri* and for 48 h at 28 °C for *A. castellanii* and at the end of incubation 25 μL of CellTiter-Glo Luminescent Cell Viability Assay (Promega) were added in each well of the 96-well plates to induce cell lysis. The resulting ATP-bioluminescence of the trophozoites was measured at room temperature using an Envision plate reader from PerkinElmer. Compounds that demonstrated >70% inhibition at 10 μM were retested following the protocol mentioned above, but with the following modifications: for the 8 or 16-point dose response curve, compounds were tested in triplicate at concentrations ranging from 0.0015 μM to 50 μM; the assay was performed in 96-well plates in 100 μL volume with 10,000 *N. fowleri* trophozoites and 5000 *A. castellanii* trophozoites. All experiments were performed in triplicate in three independent experiments (biological replicates).

*Balamuthia*. For the initial 8-point dose response study, drug testing was performed by seeding 30 μL of trophozoites (3000 parasites/well) into 384-well opaque white plates and incubating with drug or control at 37 °C for 72 h. Viability was assayed using a CellTiter-Glo assay (Promega) as previously described (Laurie et al., 2018). The ATP-bioluminescence of the trophozoites was measured at room temperature using a Tecan infinite M1000 PRO. We plated *B. mandrillaris* trophozoites in assay plates containing the compounds in 0.5 μL of DMSO to final concentrations ranging from 0.51 μM to 66 μM (8-point, 2-fold serial dilution). For the confirmation and follow up assays, we performed dose response experiments in triplicate in trophozoites, with compound concentrations ranging from 3 nM to 50 μM (15-point, 2-fold serial dilution). 0.5% DMSO was used as a negative control and 50 μM of nitroxoline (Laurie et al., 2018) was used as a positive control. All experiments were performed in triplicate with three biological replicates.

### 2.3 EC_50_ calculation

In order to calculate the EC_50_, the percent inhibition relative to maximum and minimum reference signal controls was calculated using the formula:

% Inhibition = [(mean of Maximum Signal Reference Control–Experimental Value)/(mean of Maximum Signal Reference Control–mean of Minimum Signal Reference Control)] × 100

The relative dose response data in triplicate in three independent biological replicates were exported to GraphPad Prism software 8.0 for EC_50_ calculations and statistical analysis.

### 2.4 Killing kinetics of *Balamuthia* and *Naegleria*

In order to identify if compounds have different killing kinetics, we tested the effect of promising compounds at different time points on cultures of *B. mandrillaris* and *N. fowleri*. We selected thirteen compounds for *Balamuthia*, namely auranofin, bortezomib, plicamycin, panobinostat, ponatinib, TG02, sapanisertib, GDC-0084, PF-04691502, bimiralisib, CUDC-907, latrunculin B and omipalisib, and seven for *Naegleria* namely plicamycin, panobinostat, lestaurtinib, midostaurin, bardoxolone methyl, CUDC-907 and quisinostat. We also determined the killing kinetics of amphotericin B (Amphotericin B solubilized, Sigma-Aldrich), a reference drug for PAM. Compound selection was based on CNS-permeability, clinical development (either FDA approved or clinical phase II) and potency. All compounds were tested in biological triplicate at a concentration which is two times the EC_50_ concentration. Trophozoites of *N. fowleri* or *B. mandrillaris* (10,000 in 100 μL) were treated with the compounds at two times of 48-h EC_50_ concentration in 96-well plates for 8 h, 24 h and 48 h and viability of *N. fowleri* trophozoites was measured with CellTiter-Glo Luminescent Cell Viability Assay (Promega) and *B. mandrillaris* viability was determined after incubation with fluorescein diacetate for 30 min, fixing the trophozoites with 4% PFA and measuring fluorescence using a Tecan Infinite M1000 pro fluorometer following incubation. The percentage viability of trophozoites treated with different compounds at different time points was calculated. 0.5% DMSO was used as a negative control and 50 μM amphotericin B or 15.6 μM nitroxoline (Laurie et al., 2018) were used as a positive control for *N. fowleri* and *B. mandrillaris*, respectively.

### 2.5 Activity against *Balamuthia* mature cysts

*Balamuthia* cysts were generated by adding 12 mL of galactose 20% to 8 mL of logarithmic phase trophozoite cultures and incubated at 37 °C for three days. The cysts were then plated at a density of 10,000 parasites in 100 μL per well in 96 well white plates. The compounds were added in a concentration that is 2 or 4 times the EC_50_. After 72 h of incubation, 50 μL per well of cell of CellTiter-Glo Luminescent Cell Viability Assay (Promega) were added, and Luminescence was measured using a Tecan Infinite M1000 PRO. The percentage viability of cysts treated with different compounds at different time points was calculated. 0.5% DMSO was used as a negative control, and nitroxoline and pentamidine were used as positive controls.

### *2.6* *Balamuthia* encystment response

Nitroxoline was previously shown to stimulate *Balamuthia* trophozoites to encyst. To determine if our priority compounds induce encystment, we evaluated the number of cysts present after treatment with the compounds. We plated 50,000 *Balamuthia* trophozoites in 500 μL media per well in 24 well plates and incubated at 37 °C for 72 h. The compound-treated parasites were fixed by adding 500 μL of 8% PFA and cysts were counted using a hemocytometer. Cysts and trophozoites can be differentiated morphologically under light microscopy. Results are expressed as number of cysts per mL.

### *2.7* *Balamuthia* recrudescence

In order to understand if the parasite can recover growth after drug treatment, we performed recrudescence assays adapted from a previous study (Laurie et al., 2018). *B. mandrillaris* trophozoites (10^5^ cells in 4 mL of Cerva's medium) were exposed to selected compounds at a concentration which is two times the EC_50_ for 72 h at 37 °C. In parallel, HFF cells were seeded at 5 × 10^4^ per well in four 24-well plates and incubated for 72 h at 37 °C. Compound-treated *B. mandrillaris* parasites were centrifuged, washed once with PBS and resuspended in 4 mL of complete DMEM media. 0.5 mL of resuspended parasites were transferred in duplicate to each well of a plate containing HFF. Plates were fixed with 4% PFA after 3, 5, 7 and 9 days. The percent of the HFF monolayer lysed was observed using light microscopy and recorded for each timepoint. 0.5% DMSO was used as a negative control and 15.6 μM nitroxoline (Laurie et al., 2018) was used as a positive control. All compounds were tested in three independent replicates.

## 3 Results

### 3.1 ReFRAME library compounds tested against free-living amebae *Naegleria*, *Acanthamoeba* and *Balamuthia*

**Fig. 1:**
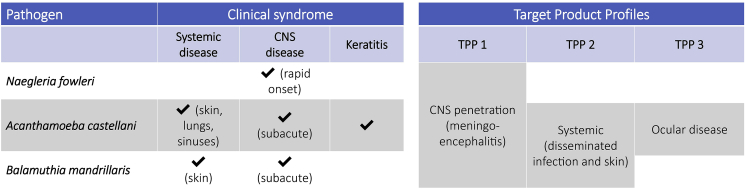
Free-living amebae infection clinical syndromes and suitable target product profile. Panel showing the possible clinical syndromes that can emerge from infection with the three free-living amebae (left) and the three possible target product profiles to guide drug development against these parasites (right).

**Fig. 2:**
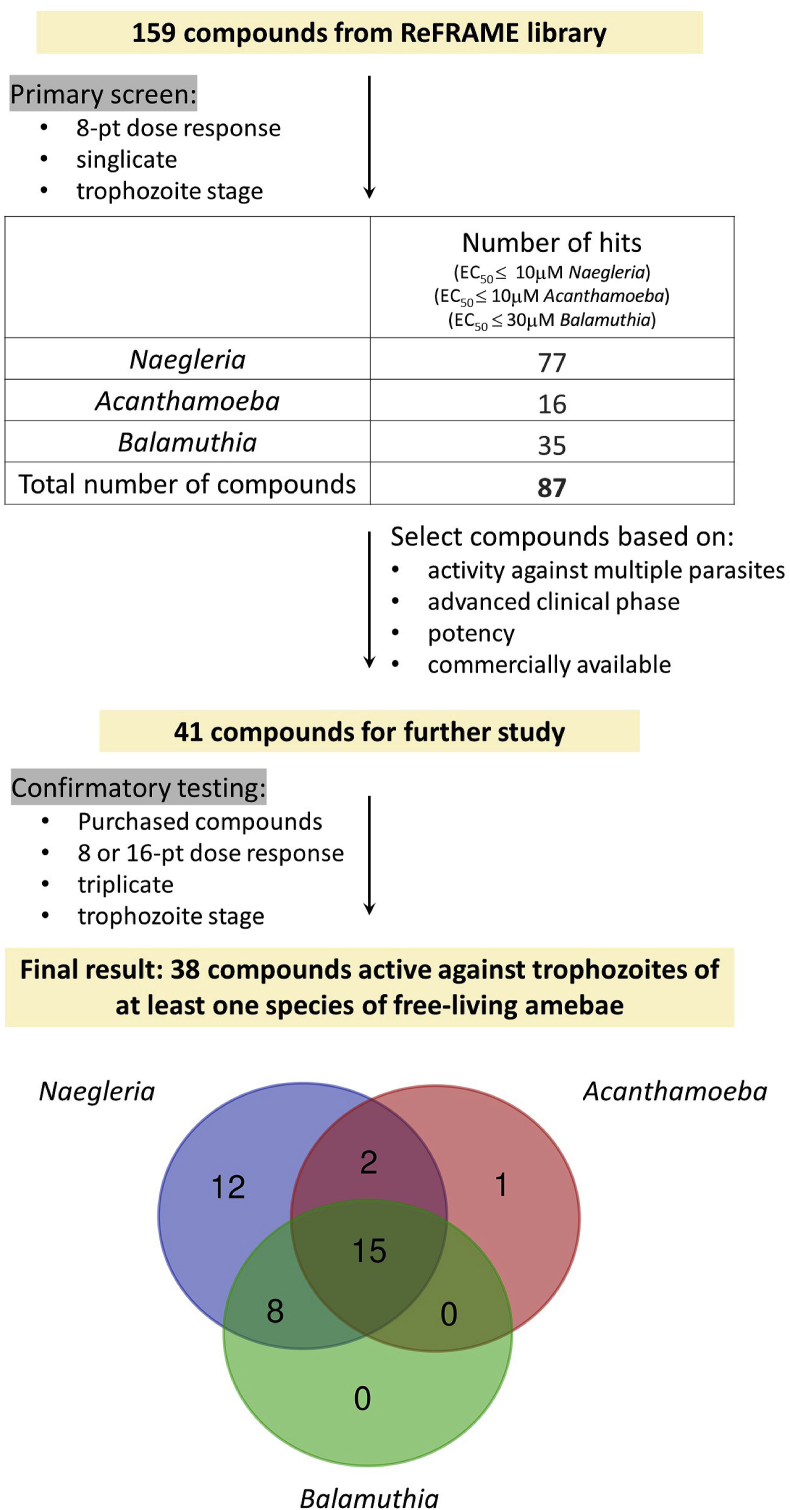
Schematic of workflow. The figure depicts the screening workflow against trophozoites of three species of free-living amebae, *N. fowleri*, *A. castellanii* and *B. mandrillaris*. The primary screen was performed with 159 compounds from the ReFRAME library (Calibr, 2019) in an 8-point dose response curve in singlicate. The number of hits obtained at the primary screen against each parasite, with an EC_50_ cutoff of 10 μM for *Naegleria* and *Acanthamoeba* and 30 μM for *Balamuthia*, are shown. Based on the described criteria, a total of 41 compounds were selected (38 from the 87 hits of the primary screen plus three additional compounds) for confirmatory tests. In the final results we obtained 38 active compounds against at least one free-living amebae. The Venn diagram shows the number of hits active against each parasite and the overlapping activity.

Improved treatment regimens are needed for *N. fowleri*, *Balamuthia* and *Acanthamoeba* infections. The parasites cause multiple clinical syndromes and the target product profiles (TPP) needed for each syndrome have unique features (Fig. 1). For the purposes of this current work, we prioritized the target product profile needed to treat infections of the CNS. We tested a subset of compounds from the ReFRAME library using the workflow in Fig. 2. The ReFRAME library was strategically designed with 11,948 known compounds that are already FDA approved or have been tested for clinical safety (mature leads or repurposing) (Janes et al., 2018). We tested a 159-compound subset of this library against the three free-living amebae. These 159 compounds were identified as active against the parasitic ameba *E. histolytica* in a single-point screen (all data available at ReFRAME online portal https://reframedb.org/#/) (Kangussu-Marcolino et al., Manuscript in preparation). We previously identified a high overlap between compounds active against the amebae *E. histolytica* and *N. fowleri* (data not shown; Ehrenkaufer et al., manuscript in preparation) leading us to investigate whether compounds active against the three free-living amebae could be identified within the 159-compound set.

The 159 compounds were tested in a single 8-point dose response curve against *N. fowleri, A. castellanii* and *B. mandrillaris* and resulted in 77 hits for *N. fowleri* (defined as EC_50_ ≤ 10 μM), 16 hits for *A. castellanii* (EC_50_ ≤ 10 μM), and 35 hits for *B. mandrillaris* (EC_50_ ≤ 30 μM) (Calibr, 2019) (Supplementary Table 1). Because of the large number of compounds in the subset of ReFRAME library, the present study was conducted with only one strain each of *Naegleria*, *Acanthamoeba* and *Balamuthia*. Since there is no single available drug that works well against *Balamuthia*, we set a higher EC_50_ cut off (≤30 μM) for *Balamuthia* than *Naegleria* or *Acanthamoeba*. Using these criteria, we obtained a list of 87 unique compounds active against at least one of the three free-living amebae. This confirms the results we have previously noted, that compounds active against *E. histolytica* are often active against *Naegleria* (Ehrenkaufer et al., manuscript in preparation). Additionally, it has been noted that compounds such as miltefosine or auranofin have activity against multiple parasitic pathogens (Aichelburg et al., 2008; Cope et al., 2016; Debnath et al., 2013; Polat et al., 2012). Whether this reflects common drug targets or some cellular aspect unique to the amebic pathogens is not known. These compounds represent the first large set of inhibitors against these highly fatal diseases. In addition to candidates for drug treatment of free-living amebae infections, these data may give insights into biological pathways that may be good drug targets in these free-living amebae.

### 3.2 Defining activity profiles of compounds against the free-living amebae *Naegleria*, *Acanthamoeba* and *Balamuthia*

In order to validate and definitively calculate EC_50_s, we selected 41 compounds for further testing. These compounds included 38 hits identified from the ReFRAME library and three compounds that are analogs of hits or which share a common target. The compounds were purchased from vendors and tested in triplicate in an 8 or 16-point dose response curves ranging from 1.5 nM to 50 μM against trophozoites of *N. fowleri, A. castellanii* and *B. mandrillaris* (Supplementary Tables 2 and 3). We also considered information relevant for clinical development, including whether the molecule has been tested in clinical trials, has CNS penetration, and the presumptive target in human cells.

Overall, we found 38 compounds active against at least one free-living amebae, 15 compounds active against all three amebae and 25 compounds active against at least two amebae (Fig. 2 and Table 1, Table 2, Table 3, Table 4). Of interest, *Naegleria* had the greatest drug susceptibility to the compounds tested, with both the most inhibitors (37 compounds) identified as well as having the most potent (low nanomolar EC_50_) inhibitors (4 compounds). Whether this reflects a biological overlap with susceptibility to *Entamoeba* (since the compounds were initially identified as targeting *Entamoeba*), a faster doubling time of *Naegleria* leading to higher drug susceptibility, inability of *Naegleria* to easily convert to the resistant cyst form *in vitro*, or some other cellular factor specific to *Naegleria* is not known. Among the two remaining free-living amebae, *Balamuthia* had more drug susceptibility (23 compounds) than *Acanthamoeba* (18 compounds). The overall positive results, including identification of high-value compounds that have late-stage clinical use data, highlights the benefit of using the ReFRAME compound library as a starting point in our drug discovery efforts. Furthermore, the data reveal that the premise of compounds targeting multiple free-living amebae is valid as 66% of the compounds with confirmed activity were active against multiple amebic species. This is the first identification of a significant number of compounds that have proven efficacy against multiple free-living amebae and is an important advance. Previous efforts have identified miltefosine with activity against *Naegleria* and *Acanthamoeba*; the compound is also used against Leishmaniasis (accessdata.fda.gov). Additionally, auranofin has been shown to be active against a wide range of parasites, including *Entamoeba*, *Giardia* and *Naegleria* (Capparelli et al., 2017; Peroutka-Bigus and Bellaire, 2018). Other compounds with activity against more than one free-living ameba include pentamidine (active against *Balamuthia* and *Acanthamoeba* (Duma and Finley, 1976; Dunnebacke et al., 2004) and corifungin (active against *Naegleria* and *Acanthamoeba* (Debnath et al., 2012b; Debnath et al., 2014). However, to date no drug candidate has been identified with potent growth inhibition effects against all three species.

**Table 1:** Compounds with activity against three free-living amebae. The compounds identified in confirmatory tests to be active against all three free-living amebae. The positive controls for each free-living ameba used in the assays *in vitro* are listed. Activity is listed as the EC_50_ in μM. NA = no activity. Compound name followed by an asterisk (*) indicates additional compounds not identified in the primary screen. All other molecules were identified from the 159 compounds of the ReFRAME library. The highest phase of clinical studies achieved with each compound and the target in humans of each compound are listed (Calibr, 2019; ClinicalTrials.gov; FDA, 2019a, Orphan drug listing; Wagenlehner et al., 2014). CNS indicates if the compound has been tested for CNS conditions (Y: yes, blank: no). The total number of compounds in this category, the number of compounds in advanced clinical phase (clinical III and higher) and the number of compounds potentially CNS penetrant are listed.

Compound summary	Compound name	Naegleria (EC_50_ μM)	Acanthamoeba (EC_50_ μM)	Balamuthia (EC_50_ μM)	Other information		
Positive controls	Miltefosine	48.3			Current standard of care for PAM; not highly effective		
	Amphotericin	0.2					
	PHMB		9.2		Current standard of care for keratitis Not effective for GAE		
	Chlorhexidine		2				
	Nitroxoline			7.8	In vitro activity against Balamuthia (Laurie et al., 2018)		
							
	Compound name	Naegleria (EC50 μM)	Acanthamoeba (EC50 μM)	Balamuthia (EC50 μM)	Target	Highest Phase	CNS
	Plicamycin	5.1	6.1	11.3	DNA	FDA Approved	Y
	Ponatinib	3.7	1.6	0.3	Bcr-Abl TK	FDA Approved	
	Milciclib maleate	8.6	8.7	7.5	CDK, TrKA	FDA orphan (Clinical II)	
	Taselisib	11.6	24.9	7.7	PIK3	Clinical III	
	TG-02	1.7	2.5	1.4	CDKs, JAK2 and FLT3	Clinical II	Y
Total: 15	PF-04691502	6.6	5.3	2.1	mTOR	Clinical II	
	Latrunculin B	0.03	1.1	0.5	Actin	Clinical I	
Advanced clinical	AZD-8835	~6.1	24.8	16.5	PI3K	Clinical I	
phase: 5	JNJ-16241199	0.03	7.2	2.7	HDAC	Clinical I	
	SB-2343	3.8	16.2	1	mTOR/PI3K	Clinical I	
CNS penetration: 2	PF-03814735	1.9	10	23.6	Aurora kinase	Clinical I	Y
	Omipalisib	1	0.1	0.02	mTOR/PI3K	Clinical I	
	Nitroxoline*	~1.5	11.2	7.8	multiple	Approved in Europe	
	SU-9516	2.3	2.8	5.1	CDK	Clinical I	
	Staurosporine	0.04	1	2.7	multi kinase	Preclinical

**Table 2:** Compounds with activity against two free-living amebae. The compounds identified in confirmatory tests to be active against two of the three free-living amebae. The positive controls for each free-living ameba used in the assays *in vitro* are listed. Activity is listed as the EC_50_ in μM. NA = no activity. Active^1^ = Auranofin activity previously reported: 100% killing at 8.8 μM with 72 h treatment (Peroutka-Bigus and Bellaire, 2018). Active^2^ = Bortezomib activity previously reported: IC_50_ of 0.6 μM with 72 h treatment (Colon et al., 2019). All compounds were identified from the 159 compounds of the ReFRAME library. The highest phase of clinical studies achieved with each compound and the target in humans of each compound are listed (Calibr, 2019; ClinicalTrials.gov; FDA, 2019a, Orphan drug listing). Prescription^H^ indicates historical use of the compound as prescription drug (Calibr, 2019). CNS indicates if the compound has been tested for CNS conditions (Y: yes, blank: no). The total number of compounds in this category, the number of compounds in advanced clinical phase (clinical III and higher) and the number of compounds potentially CNS penetrant are listed.

Compound summary	Compound name	Naegleria (EC_50_ μM)	Acanthamoeba (EC_50_ μM)	Balamuthia (EC_50_ μM)	Other information		
Positive controls	Miltefosine	48.3			Current standard of care for PAM; not highly effective		
	Amphotericin	0.2					
	PHMB		9.2		Current standard of care for keratitis Not effective for GAE		
	Chlorhexidine		2				
	Nitroxoline			7.8	In vitro activity against Balamuthia (Laurie et al., 2018)		
							
	Compound name	Naegleria (EC50 μM)	Acanthamoeba (EC50 μM)	Balamuthia (EC50 μM)	Target	Highest Phase	CNS
Total:	Lestaurtinib	0.4	19.2		FLK, TrK, JAK	FDA orphan (Clinical III)	Y
Acanthamoeba and Naegleria: 2	CHROMOMYCIN A3	2.1	2.7		DNA	Prescription H	

Balamuthia and Naegleria: 8							
	Auranofin	8.8 Ref1		17.2	5-LO	FDA Approved	Y
	Panobinostat	0.5		8.8	HDAC	FDA Approved	Y
	Bortezomib	0.6 Ref2		14.5	proteasome	FDA Approved	Y
	Astemizole	10.7		15.8	HH1R	FDA Withdrawn	
Advanced clinical	CUDC-907	0.03		1.3	HDAC, PI3K	Clinical II	
phase: 5	Quisinostat	0.7		6.1	HDCA	Clinical II	
	Sapanisertib	4		6.8	mTOR	Clinical II	Y
CNS penetration: 5	NVP-BGT226	8.9		1.2	mTOR/PI3K	Clinical II

**Table 3:** Compounds with activity against one free-living ameba. The compounds identified in confirmatory tests to be active against one of the three free-living ameba species. The positive controls for each free-living ameba used in the assays *in vitro* are listed. Activity is listed as the EC_50_ in μM. NA = no activity. Compound name followed by an asterisk (*) indicates additional compounds not identified in the primary screen. All other molecules were identified from the 159 compounds of the ReFRAME library. The highest phase of clinical studies achieved with each compound and the target in humans of each compound are listed (Calibr, 2019; ClinicalTrials.gov; FDA, 2019a, Orphan drug listing). Prescription^H^ indicates historical use of the compound as prescription drug (Calibr, 2019). CNS indicates if the compound has been tested for CNS conditions (Y: yes, blank: no). The total number of compounds in this category, the number of compounds in advanced clinical phase (clinical III and higher) and the number of compounds potentially CNS penetrant are listed.

Compound summary	Compound name	Naegleria (EC50 μM)	Acanthamoeba (EC50 μM)	Other information		
Positive controls	Miltefosine	48.3		Current standard of care for PAM; not highly effective		
	Amphotericin	0.2				
	PHMB		9.2	Current standard of care for keratitis Not effective for GAE		
	Chlorhexidine		2			
						
	Compound name	Naegleria (EC50 μM)	Acanthamoeba (EC50 μM)	Target	Highest Phase	CNS
Total:	Midostaurin*	0.5		multi kinase	FDA approved	
Naegleria: 12	Telotristat etiprate	3.9		TPH1	FDA Approved	
	Lomitapide	7.1		MTTP	FDA Approved	
	Vosaroxin	3.5		DNA topo	FDA orphan (Clinical III)	
Acanthamoeba: 1	Alvocidib	3.5		CDK9	FDA orphan (Clinical III)	
	Bardoxolone methyl	0.3		multiple	FDA orphan (Clinical III)	
	Clemizole*	24.4		HH1R	Clinical II/Prescription H	
Advanced clinical	Cycloheximide	5.3		GSK-3beta	Prescription H	
phase: 6	CC-115	8.5		mTOR and DNA-PK	Clinical II	Y
	AZD-5438	5.7		CDK1/2	Clinical I	
CNS penetration: 1	LY-2874455	2.6		FGFR	Clinical I	
	TUBERCIDIN	1.4		DNA Pol	Prescription H	
	Ethynylcytidine		1.7	RNA Pol	Clinical II

**Table 4:** mTOR and PI3K inhibitors and their activity against free-living amebae. Inhibitors of mTOR and PI3K with distinct selectivity were tested in 8 or 16-point drug response curves against trophozoites of *Naegleria*, *Acanthamoeba* and *Balamuthia*. The table is divided in categories of selectivity to mTOR, PI3K or dual mTOR/PI3K inhibitors. The potency of each molecule against the purified human protein is represented in the following scale: +++++ (<1 nM), ++++ (1 nM < 5 nM), +++ (5 nM < 10 nM), ++ (10 nM < 50 nM), + (>50 nM) (Barlaam et al., 2015; Beaufils et al., 2017; Edwards and Wandless, 2007; Garcia-Echeverria and Sellers, 2008; Hart et al., 2013; Heffron et al., 2016; Hsieh et al., 2012; Knight et al., 2010; Maira et al., 2008; Markman et al., 2012; Mateo et al., 2017; Mortensen et al., 2015; Ndubaku et al., 2013; Qian et al., 2012; Sarkaria et al., 1998; Sedrani et al., 1998; Shor et al., 2008; Sun, 2013; Sutherlin et al., 2011; Venkatesan et al., 2010; Yano et al., 1993; Yuan et al., 2011). Activity against free-living ameba trophozoites are shown with the EC_50_ in μM. NA = no activity. Compound name followed by an asterisk (*) indicates additional compounds not identified in the primary screen. All other molecules were identified from the 159 compounds of the ReFRAME library. The highest phase of clinical studies achieved with each compound and the target in humans of each compound are listed. CNS indicates if the compound has been tested for CNS conditions (Y: yes, blank: no).

Selective to:	Compound name	mTOR	PI3K	Naegleria (EC_50_ μM)	Acanthamoeba (EC_50_ μM)	Balamuthia (EC_50_ μM)	Highest Phase	CNS
mTOR	Sapanisertib	++++	+	4	NA	6.8	Clinical II	Y
	Gedatolisib*	++++	+	NA	~12.1	16.4	Clinical II	
	Vistusertib*	++++		NA	NA	9.3	Clinical II	
	CC-115	++	+	8.5	NA	NA	Clinical II	Y
PI3K	Taselisib		+++++	11.6	24.9	7.7	Clinical III	
	GDC-0084*	+	+++	3.7	18	3.7	Clinical II	Y
	NVP-BGT226		++	8.9	NA	1.2	Clinical I/II	
	AZD-8835		+	~6.1	24.8	16.5	Clinical I	
	Wortmannin*		+++	15.7	NA	NA	Clinical	
	GSK-2636771*		+++	NA	NA	NA	Clinical I/II	
	XL765*	+	++	NA	NA	NA	Clinical II	
Dual	Omipalisib	+++++	+++++	1	0.1	0.02	Clinical I	
	SB-2343	++++	+++	3.8	16.2	1	Clinical I	
	Dactolisib	+++	++	NA	NA	1.7	Clinical II	
	Bimiralisib*	++	+++	14.4	NA	6.3	Clinical II	Y
	PF-04691502	++	++++	6.6	5.3	2.1	Clinical II	Y
	Apitolisib*	++	+++	6.1	NA	NA	Clinical II	
Rapalogs	Rapamycin*	++++		NA	NA	NA	FDA approved	
	Everolimus*	++++		NA	NA	NA	FDA approved	
	Temsirolimus*	++++		NA	NA	NA	FDA approved	

### 3.3 Compounds with activity against all three free-living amebae

Several of the confirmed hits inhibited the growth of all three amebae, *N. fowleri*, *A. castellanii* and *B. mandrillaris* (Table 1). A total of 15 active compounds were identified from the ReFRAME screen, of which plicamycin and ponatinib are FDA approved. CNS-penetrant plicamycin (Walker et al., 1976) is equipotent against *Naegleria* and *Acanthamoeba* with EC_50_ of 5–6 μM. It exhibits similar activity to PHMB, the current standard of care for *Acanthamoeba* keratitis, and about 10-fold more activity than miltefosine used for *Naegleria* infection. While plicamycin had less activity against *Balamuthia*, another FDA approved drug ponatinib showed the highest potency against *Balamuthia* among all three organisms, with an EC_50_ of 0.3 μM. Ponatinib was 25-fold more potent than the control compound nitroxoline used in the study against *Balamuthia.* Ponatinib was equipotent to chlorhexidine, another standard of care for *Acanthamoeba*. It was equally active against *Naegleria* with an EC_50_ of 3.7 μM. Our study further identified a phase III compound taselisib as active against all three parasites, but potency was less than plicamycin or ponatinib. We identified three phase II compounds, milciclib maleate, TG02, and PF-04691502, as active against all three parasites. Of these, CNS-permeable (Pasha et al., 2012) TG02 exhibited low micromolar potency against all three pathogens. Six compounds which are in phase I clinical trials showed activity against all three parasites; among those omipalisib was the most potent against all three parasites and the most potent compound overall against *Acanthamoeba* with an EC_50_ of 100 nM. Latrunculin B was also highly active against all three parasites, being especially potent against *Naegleria* with an EC_50_ of 0.03 μM.

### 3.4 Compounds with activity against two free-living amebae

A total of 10 compounds were identified from ReFRAME screen that target at least two amebae (Table 2). Of these, two were active against *Naegleria* and *Acanthamoeba* and eight were active against *Naegleria* and *Balamuthia*. Among the compounds active against both *Naegleria* and *Acanthamoeba*, CNS-permeable lestaurtinib is more clinically relevant, as it is in phase III clinical studies (NCT00557193) and has received orphan designation from the FDA (FDA, Orphan drug listing). It displays a good potency with EC_50_ of 0.4 μM against *Naegleria*. Among the eight compounds that were found active against *Naegleria* and *Balamuthia*, three (auranofin, panobinostat and bortezomib) are FDA-approved. The anti-*Naegleria* activity of auranofin and bortezomib has been previously noted; these two compounds were identified as hits against *Naegleria* in two separate studies (Colon et al., 2019; Peroutka-Bigus and Bellaire, 2018). Four compounds (CUDC-907, quisinostat, sapanisertib and NVP-BGT226) which were in phase II clinical trials showed activity against both *Naegleria* and *Acanthamoeba*, but only sapanisertib is likely to be CNS penetrant. Sapanisertib is equipotent to nitroxoline against *Balamuthia* with an EC_50_ of 6.8 μM and 12-fold more potent than miltefosine against *Naegleria*.

### 3.5 High-value inhibitors of *Naegleria*

A total of 37 inhibitors of *Naegleria* were identified from ReFRAME library screen (Table 1, Table 2, Table 3). Two (auranofin and bortezomib) were previously studied; we obtained the EC_50_ of the remaining 35 inhibitors. All compounds had better activity than the current standard of care miltefosine. Six compounds (plicamycin, TG02, lestaurtinib, panobinostat, CC-115 and sapanisertib) have known CNS penetration. Lestaurtinib and panobinostat displayed nanomolar potency and they are either in late stage of development or FDA approved providing an opportunity of repurposing for the treatment of PAM. Of the compounds active against *Naegleria* only, three (vosaroxin, alvocidib and bardoxolone methyl) are in phase III clinical trials and have received orphan drug designation, and other three (midostaurin, telotristat etiprate and lomitapide) have been approved by the FDA. Of special interest is midostaurin, a derivative of staurosporine, which was noted to have high efficacy against *Naegleria* (EC_50_ 0.6 μM) (Table 3). While staurosporine had activity against all three species, its use for clinical syndromes is limited due to lack of clinical trial data. However, its derivative midostaurin is FDA approved. Overall, a number of these compounds offer important potential advantages over the current therapies: higher potency and known CNS penetration which may prove beneficial in management of *Naegleria* PAM. The late stage or FDA approved status of the compounds we identified means that transition to clinical use could be rapidly facilitated.

### 3.6 mTOR and PI-3 kinase targets are frequent in the set of compounds active against the free-living amebae

In order to determine whether conserved pathways represent important new targets against the free-living amebae, we analyzed the list of targets in humans of the studied compounds. Of the 159 compounds tested against the three parasites, there were 16 compounds that target mTOR and/or PI3 kinases; eleven of these compounds were identified in the primary screen (Supplementary Table 1) and nine compounds were subsequently confirmed active against at least one parasite (Table 4).

To understand if the potency or enzyme selectivity of these compounds against the human targets could be related to the activity found against the free-living amebae, we expanded the panel of mTOR/PI3K inhibitors. We tested 11 additional compounds of this class, with diverse chemical structures, for their activity against trophozoites of all three free-living amebae (Table 4). The mTOR/PI3K panel was organized in four categories according to the compound selectivity to the human target: mTOR, PI3K, dual mTOR/PI3K activity or rapamycin analogs (rapalogs) and the potency against the human target is shown in Table 4.

Five of the additional inhibitors are active at least against one parasite. The category of dual inhibitors presented slightly higher activity on the free-living amebae followed by the selective inhibitors of mTOR or PI3K, and the potency of most the compounds was higher against *Balamuthia* than *Naegleria* or *Acanthamoeba*. Of note, none of the rapalogs was active against any of the parasites. Rapalogs bind to FKBP12 and the complex inhibits mTOR by blocking substrate access (Yang et al., 2013), a different mechanism than the PI3K or mTOR selective inhibitors, which compete with ATP for the binding pocket of these kinases (Zheng and Jiang, 2015). Based on our results, compounds which selectively bind the ATP-binding site of human mTOR and PI3K are of higher interest for activity against the free-living amebae.

Our strategy identified two additional CNS penetrant experimental compounds GDC-0084 and bimiralisib. GDC-0084 is active against all three parasites and exhibited similar potency against both *Naegleria* and *Balamuthia* with an EC_50_ of 3.7 μM. This is two-fold more active than nitroxoline against *Balamuthia* and 13-fold more effective than miltefosine against *Naegleria*. Inhibitors of the mTOR pathway have been shown to be active against *Plasmodium falciparum*, *Trypanosoma brucei*, *T. cruzi*, *Leishmania major*, and *L. donovani*, highlighting this class of compounds as possible candidates for the treatment of parasitic infections (Diaz-Gonzalez et al., 2011; Khadir et al., 2018; Mott et al., 2015). Our study is the first to demonstrate that mTOR inhibitors are active against amebic parasites.

### 3.7 Variable killing kinetics of compounds active against free-living ameba

**Fig. 3:**
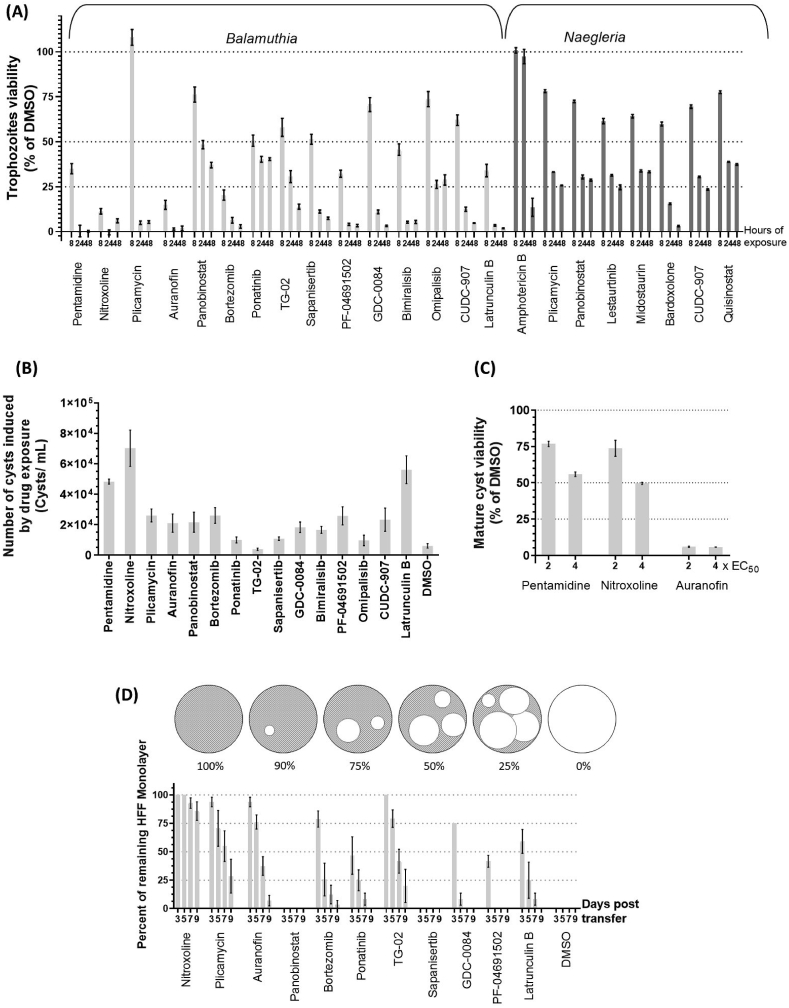
Phenotypic profile of priority compounds against *Balamuthia* and *Naegleria*. (A) Killing kinetics in *Balamuthia* and *Naegleria* trophozoites. Graph showing the % of viability of *Balamuthia* and *Naegleria* trophozoites assessed *in vitro* at 8, 24 and 48 h of drug treatment using 2 times the following EC_50_ concentrations: for *Balamuthia*: nitroxoline 7.8 μM (positive control), plicamycin 11.3 μM, auranofin 17.2 μM, panobinostat 8.8 μM, bortezomib 14.5 μM, ponatinib 0.3 μM, TG02 1.4 μM, sapanisertib 6.8 μM, GDC-008 3.7 μM, PF-04691502 2.1 μM, bimiralisib 6.3 μM, omipalisib 0.02 μM, CUDC-907 1.3 μM, latrunculin B 0.5 μM; and for *Naegleria*: amphotericin B 0.4 μM (positive control), plicamycin 5.1 μM, Panobinostat 0.5 μM, lestaurtinib 0.4 μM, midostaurin 0.5 μM, bardoxolone methyl 0.3 μM, CUDC-907 0.03 μM, quisinostat 0.7 μM. *Naegleria* viability was assessed by measuring luminescence after incubation with CellTiter-Glo assay (Promega). *Balamuthia* viability was assessed by measuring fluorescence after incubation with the vital dye fluorescein diacetate at the defined time point. Treatment with DMSO at each time point was considered 100% of viability. Values are expressed as mean ± standard error. (B) Encystment response of *Balamuthia*. Graph showing the number of cysts formed after treatment of *Balamuthia* trophozoites with 2 times the EC_50_ concentrations for 72 h. EC_50_ values for the drugs used in this assay are listed above. Number of cysts are expressed as mean ± standard error. (C) *Balamuthia* mature cyst viability after compound treatment. Graph showing the percent of *Balamuthia* cyst viability assessed *in vitro* at 72 h of drug treatment using 2 or 4 times the EC_50_ concentrations which is indicated at the x-axis with number 2 and 4 (2xEC_50_ or 4xEC_50_). Treatment with DMSO at each time point was considered 100% of cyst viability. Values are expressed as mean ± standard error. (D) Recrudescence of *Balamuthia* after drug treatment. Graph showing percent of remaining HFF monolayer at days 3, 5, 7, and 9 post transfer of *Balamuthia* parasites previously treated for 72 h at a concentration which is two times the following EC_50_ concentrations: nitroxoline 7.8 μM (positive control), plicamycin 11.3 μM, auranofin 17.2 μM, panobinostat 8.8 μM, bortezomib 14.5 μM, ponatinib 0.3 μM, TG02 1.4 μM, sapanisertib 6.8 μM, GDC-008 3.7 μM, PF-04691502 2.1 μM, latrunculin B 0.5 μM. DMSO was used as negative control. Integrity of HFF monolayer was assessed by light microscopy. Schematic represents HFF monolayer (gray stripes) and lysis (white). As lysis promoted by the recrudescent trophozoite increases, the percent of remaining HFF monolayer decreases. Values are expressed in mean ± standard error.

Since these CNS-invasive parasites lead to rapid and extensive damage in the brain, identification of fast-acting drugs that can rapidly kill the ameba present in the brain would be highly desirable. To demonstrate if CNS-permeable and additional potent compounds can rapidly kill, we performed a growth inhibition study at 8 h, 24 h and 48 h of treatment using 2x EC_50_ concentrations (Fig. 3A). For *Balamuthia* we tested plicamycin, auranofin, panobinostat, bortezomib, ponatinib, TG02, sapanisertib, GDC-0084, bimiralisib, PF-04691502, omipalisib, CUDC-907 and latrunculin B. Pentamidine and nitroxoline were used as positive controls. With *Naegleria*, we tested plicamycin, panobinostat, lestaurtinib, midostaurin, bardoxolone methyl, CUDC-907, and quisinostat. Amphotericin B was used as a positive control. We compared the growth inhibition of compound-treated trophozoites at different time points with that of the DMSO-treated control.

Growth inhibition study on *B. mandrillaris* demonstrated an inhibitory effect of auranofin and bortezomib as early as 8 h post-exposure (Fig. 3A). Thus, the overall effect of auranofin and bortezomib was relatively fast and comparable to nitroxoline. Plicamycin, sapanisertib, GDC-0084, bimiralisib, PF-04691502, CUDC-907 and latrunculin B, all demonstrated pronounced effect on cell viability at 24 h, which is comparable to pentamidine, which is part of the standard of care for *Balamuthia* infection. TG02 had maximal killing by 48 h. Three compounds presented slower inhibition effect, panobinostat, ponatinib and omipalisib, reaching between 50 and 75% of inhibition after 48 h.

When a similar assay was performed with *N. fowleri*, we noted a more uniform behavior with most compounds reaching a maximum inhibition of about 75% at 24 h. One exception is bardoxolone methyl, which inhibited about 85% of growth at 24 h and 97% at 48 h. Although bardoxolone methyl is not CNS penetrant, this result opens the path for exploring similar compounds with better pharmacokinetics such as omaveloxolone (Reisman et al., 2019). In our study, 2x EC_50_ concentration of amphotericin B did not induce growth inhibition at 24 h but showed maximum growth inhibition at 48 h. Previous studies have explored the killing kinetic of posaconazole over *N. fowleri* where they observed ~20% inhibition after 8 h of treatment with 50 μM. After 24 h the inhibition rate reached ~90% (Debnath et al., 2017). Our results have identified more compounds with rapid killing kinetics than previously identified.

### 3.8 Latrunculin B stimulates *balamuthia* encystment

It was previously noted that treatment with certain drugs induces encystment of *B. mandrillaris* (Laurie et al., 2018). Although this likely inhibits efficient eradication of the pathogen as the cysts are known to be more resistant to drugs, encystment induction could be useful to slow down disease progression and reduce tissue damage. To verify if this feature is present among our priority compounds active against *Balamuthia*, we counted the number of cysts formed from *Balamuthia* trophozoites after 72 h of treatment with compounds at concentrations that are 2 times the EC_50_ (Fig. 3B). We noted that, besides the positive controls (pentamidine and nitroxoline), only latrunculin B efficiently induced encystment of *B. mandrillaris*. This effect can be related to the ability of latrunculin B to inhibit actin polymerization as other compounds with related function have been shown to affect encystment efficacy in *Entamoeba invadens* (Makioka et al., 2004). Plicamycin, auranofin, panobinostat, bortezomib, GDC-0084, bimiralisib, PF-04691502 and CUDC-907 showed mild induction of encystment. Ponatinib, TG-02, sapanisertib and omipalisib do not seem to induce encystment as the number of cysts found after treatment was not considerably greater than the value found for DMSO treatment. Compounds that induce cyst formation can be explored for drug development and would most likely be combined with a compound that also inhibits viability of mature cysts.

### 3.9 Auranofin is active against *balamuthia* mature cysts

Given that *Balamuthia* cysts were found in the brain of patients with GAE (Bando et al., 2012; Kum et al., 2019), a drug capable of reducing cyst viability is highly desired. To evaluate cyst inhibition, we tested our priority compounds against mature cysts. Trophozoites were induced to encyst for 3 days; at this point the encystation is highly efficient and trophozoites are largely not identified in the culture under light microscopy. We screened the activity of the compounds against these mature cysts at concentrations of 2 and 4 times the EC_50_ in multiple biological replicates. Compounds that inhibited cyst viability at 20% or greater were considered active. Auranofin was the only compound that met these criteria inhibiting 93% of viable cysts at 2 times of the EC_50_ concentration and 94.5% of cysts at 4 times of the EC_50_ concentration (Fig. 3C). This is an outstanding effect given that the positive controls exhibited much more modest activity, with about 50 and 40% of viability reduction at 4 times the EC_50_ for nitroxoline and pentamidine respectively. Drugs active against mature cysts could be an important addition to the treatment of GAE with *Balamuthia*.

### *3.10* *Balamuthia* recrudescence is delayed by auranofin, plicamycin and TG02

Drug treatment induces the free-living amebae to various phenotypes which will ultimately have the purpose of slowing down or stop host cell destruction. To evaluate how fast the trophozoites can recover from a single-dose drug treatment, we performed a recrudescence assay (Laurie et al., 2018). *Balamuthia* trophozoites were treated with each drug at 2x the EC_50_ for 72 h; the drugs were then removed, and the treated parasites were transferred to HFF monolayers. The percent of monolayer remaining was observed 3, 5, 7, and 9 days post transfer (Fig. 3D). The trophozoites treated with DMSO consistently lysed the whole monolayer one day after transfer. We tested the effects of plicamycin, auranofin, panobinostat, bortezomib, ponatinib, TG02, sapanisertib, GDC-0084, PF-04691502 and latrunculin B on *Balamuthia* recrudescence. Nitroxoline was used as positive control of delayed recrudescence. TG02, plicamycin and auranofin showed the best results having protected more than 60% of the monolayer for at least 5 days. Bortezomib, ponatinib and latrunculin B treatments had 25% of remaining monolayer at day 5. GDC-0084 showed the best delay in recrudescence among the populations treated with mTOR/PI3K inhibitors, with 75% remaining monolayer at day 3 and ~10% at day 5, while PF-04691502 had 25% and sapanisertib had no remaining of HFF monolayer at day 3. This result may suggest that mTOR/PI3K inhibitors have a transitory effect on the parasite growth. With these results we identified at least five compounds that considerably delay the recovery of *B. mandrillaris* trophozoites after a single treatment of 72 h.

## 4 Discussion

**Fig. 4:**
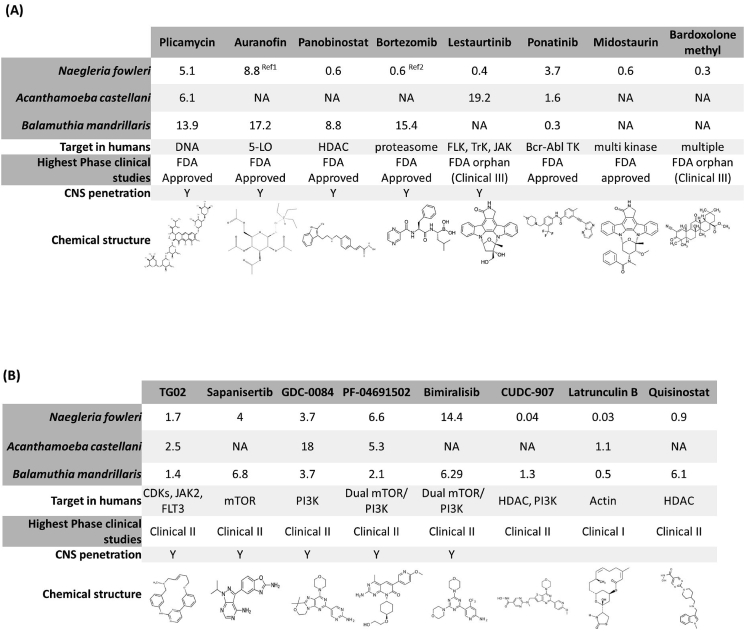
Priority compounds against free-living amebae. Panels showing a summary of properties of the compounds prioritized for TPP1, with focus on CNS penetrant compounds. Activities against free-living ameba trophozoites are shown with the EC_50_ in μM. NA = no activity. The highest phase of clinical studies achieved with each compound and the target in humans of each compound are listed. CNS penetration indicates if the compound has been tested for CNS conditions (Y: yes, blank: no). (A) Priority compounds that have received FDA approval or orphan designation. Ref^1^ = Auranofin activity previously reported: 100% killing at 8.8 μM with 72 h treatment (Peroutka-Bigus and Bellaire, 2018). Ref^2^ = Bortezomib activity previously reported: IC_50_ of 0.6 μM with 72 h treatment (Colon et al., 2019). (B) Priority compounds that are in clinical studies.

The free-living amebae *Naegleria*, *Acanthamoeba*, and *Balamuthia* cause CNS infections with meningoencephalitis and granulomatous encephalitis and high fatality rates. Additionally, *Acanthamoeba* and *Balamuthia* can cause systemic infections and *Acanthamoeba* causes severe keratitis often necessitating corneal transplant. There are currently no good treatment regimens available against these pathogens. We screened a small subset of a high value ReFRAME library and identified new candidates for a drug development pipeline for these important human pathogens. Our efforts identified 16 compounds as high priority which either have data to support CNS penetration (Lee et al., 2015; NCT02133183; NCT02942264; NCT03765983; Raedler, 2016; Walker et al., 1976) or showed sub-micromolar potency against at least one of the free-living amebae. All priority compounds have significant clinical use data and were organized in two panels: compounds with FDA approval or orphan designation (plicamycin, auranofin, panobinostat, bortezomib, lestaurtinib, ponatinib, midostaurin, bardoxolone methyl) (Fig. 4A) and compounds in clinical studies (TG02, sapanisertib, GDC-0084, PF-04691502, bimiralisib, CUDC-907, latrunculin B, quisinostat) (Fig. 4B). A few parameters of pharmacokinetics and toxicity of the 16 priority compounds available in the literature and the CC_50_ in human cell lines are included in Supplementary Tables 4 and 5 These compounds represent an important new path forward in the development of treatment algorithms for these fatal and destructive diseases.

Both plicamycin and TG02 are CNS penetrant and were active against all three parasites. Plicamycin, formerly known as mithramycin, is an aureolic acid-type antineoplastic antibiotic. It is used as an anticancer agent in the therapy of testicular cancer (Osada et al., 2013) and was previously studied for the treatment of brain tumors (Walker et al., 1976). Recent studies showed that plicamycin decreased neuronal death in mice and because of its neuroprotective effects (Osada et al., 2013) plicamycin may be a promising candidate for both *Naegleria* and *Acanthamoeba* infections. Plicamycin was not highly potent against *Balamuthia*, however, it was able to dramatically reduce parasite viability relatively rapidly and delayed the treated parasite recrudescence. Although plasma levels of plicamycin were found to reach a maximum of about 1.5 μM in human patients (Fang et al., 1992), an analog of this compound (MTM-SK) generated by genetically engineering the plicamycin biosynthetic pathway have been found to reach plasma levels as high as 20 μM in mice (Malek et al., 2012). It is worth exploring alternative approaches including combination treatments and multiple dosing for future testing of plicamycin against free-living amebae *in vivo*.

Our study identified a broad-spectrum kinase inhibitor TG02 that is active against all three amebae with a low micromolar EC_50_. TG02 is in development for multiple oncology indications including leukemia and glioblastoma (Goh et al., 2012). Extensive pre-clinical studies showed good stability in human microsomes, Caco-2 cell permeability, and low inhibition of most CYPs (Pasha et al., 2012). Most importantly, TG02 was found to accumulate in the mouse brain reaching a maximum concentration of 2.1 μM (Pasha et al., 2012), making it likely to be able to target CNS infections such as PAM and GAE. TG02 has completed Phase I trials, with oral dosing up to 300 mg, showing few major adverse effects (Hofmeister et al., 2015). Currently, the compound is in phase II trials (NCT02942264).

Auranofin is FDA approved and was repurposed for use against *E. histolytica* infection. It was previously described as active against *Naegleria* (Peroutka-Bigus and Bellaire, 2018) and was found in our study to be active against *Balamuthia* trophozoite and cyst forms. In a study describing neuroprotective properties of auranofin, the gold concentration was found to be 4.79 μM in the brain tissue and higher concentrations of gold were measured in other organs, for example about 250 μM in kidney and 150 μM in liver when auranofin was administrated orally in mice at 2 mg/kg each day for 7 days (Madeira et al., 2013). Due to its great activity against *Balamuthia* cysts, auranofin is a promising compound for treatment against brain and systemic infections with this parasite.

Given the unique epidemiological scenario associated with *Naegleria* infections (very recent exposure to fresh-water swimming and rapid progression of symptoms), drugs highly active against *Naegleria* can be prioritized for clinical use in cases of presumptive *Naegleria* PAM as imminent death is otherwise highly likely; compounds that are in late stage of clinical development and CNS penetrant would be priorities in this area. An FDA-approved BBB (blood-brain barrier) permeable compound panobinostat and another CNS permeable phase III compound lestaurtinib were particularly potent against *N. fowleri* with EC_50_ in the nanomolar range. Other compounds of similar structure, staurosporine and midostaurin, have also been identified as potent against *Naegleria*. Lestaurtinib (CEP-701) is an orally bioavailable indolocarbazole alkaloid compound that has activities against tropomyosin receptor kinases, neurotrophin receptors, FLT3, and JAK2 (Wu et al., 2018). It completed phase II clinical trials in acute myeloid leukemia and is currently in phase III clinical trial (NCT00084422). Panobinostat is a histone deacetylase inhibitor and antineoplastic agent which in a pre-clinical study with glioma models was detected in mice brainstem glioma at 0.55 μM (Hennika et al., 2017; NCT02133183). Panobinostat is FDA-approved for use in combination with bortezomib in refractory or relapsed multiple myeloma (Raedler, 2016). Since both bortezomib and panobinostat were found to be potent against *N. fowleri*, a combination therapy with panobinostat and bortezomib may hold promise for the treatment of PAM.

Four mTOR inhibitors that are under clinical investigation for oncologic conditions located in the head and brain showed micromolar EC_50_ against two or three free-living amebae (sapanisertib in glioblastoma (NCT02133183), GDC-0084 in brain metastasis (NCT03765983), PF-04691502 and bimiralisib in Head and Neck Squamous Cell Carcinoma (NCT03740100; Tonlaar et al., 2017). The identification of these mTOR inhibitors active against free-living amebae led us to investigate other mTOR or PI3 kinase inhibitors with differing chemical structures against these parasites. A dual mTOR/PI3K inhibitor PF-04691502, and a PI3K selective inhibitor GDC-0084 are of particular interest because they have better potency overall and are active against all three amebae. GDC-0084 showed better killing kinetics and delay of recrudescence in *B. mandrillaris*. In a mouse model of human glioblastoma, GDC-0084 showed good CNS penetration, reaching 5.5 μM in brain tissue at 2 h after oral administration (Salphati et al., 2016). This led to phase I and II clinical trials of GDC-0084 for the treatment of patients with breast cancer that has metastasized to the brain (NCT03765983).

So far, we have focused largely on compounds that may have CNS penetration. However, both *Acanthamoeba* and *Balamuthia* also cause systemic disease (Fig. 1) and compounds that are effective against these two parasites but without CNS penetration, such as ponatinib, omipalisib and latrunculin B, would still be very useful. Furthermore, for *Acanthamoeba* keratitis, treatment is with systemic therapy and eye drops. Thus, formulation to allow direct ocular instillation would also be an important consideration for *Acanthamoeba* keratitis (Fig. 1). A good example among our priority compounds is latrunculin B, which is potent against *Acanthamoeba* and has been previously tested as ophthalmic solution against glaucoma (Rasmussen et al., 2014).

For future studies, the efficacy of these compounds against multiple clinical strains, encysting or mature cysts of *Acanthamoeba* will be important parameters to define. These features will help identify the compounds that are of the highest priority for use in animal studies. A *Naegleria* PAM animal model is established (Debnath et al., 2012b) and can be used to identify *in vivo* efficacy of the compounds. Animal studies with combination therapies can also be used to define likely optimal therapeutic regimens. However, it is important to note that amphotericin, despite being successful to treat PAM in an *in vivo* animal model, is not efficacious in humans. But given that clinical trials for these pathogens will not be possible, *in vivo* animal studies will serve as the best available proxy.

Getting approval for new drugs is a complex and long path. In order to obtain a faster track to the market, a compound that is already approved by the FDA or has significant human use data would be ideal. Moreover, given the overall rarity of the clinical syndromes caused by the free-living amebae, clinical trials are not feasible. With demonstration of efficacy of an FDA-approved drug in an animal model and human safety use data, it is possible to include it in the treatment regimen or approach the FDA for orphan drug designation, as was done for auranofin in 2010 for the treatment of amebiasis (Debnath et al., 2013). New treatments against *Naegleria* PAM and *Acanthamoeba* and *Balamuthia* GAE would also be eligible for a fast track drug development and compassionate use by the FDA and expanded access under a treatment protocol or Investigational New Drug (IND) program (FDA, 2018, 2019b). Given that the compounds discussed above have late stage clinical use, pending proof of *in vivo* efficacy, the regulatory path forward appears promising.

Overall, our efforts identified many high-value compounds with excellent efficacy against the free-living amebae including compounds that have passed regulatory hurdles and have CNS penetration. The effort and results demonstrate the value of screening established libraries to identify new drug candidates for neglected parasitic diseases.

## Appendix A Supplementary data

Multimedia component 1 **Primary screening results.** Activity of 159 compounds from the primary screen against *Naegleria*, *Acanthamoeba* and *Balamuthia* are listed. NA is no activity. Smiles data are included. **Confirmatory test results.** Activity of 41 compounds plus 11 additional inhibitors of the mTOR pathway tested in triplicate against *Naegleria*, *Acanthamoeba* and *Balamuthia* are listed. EC_50_ (μM), values of R^2^ and efficacy are included. NT is not tested, none is “not calculated”. Sources for all the compounds. ADMET data of the priority compounds, Toxicity, Maximum plasma concentration (Cmax), time for maximum plasma concentration (Tmax) and half-life (t1/2) in mice and/or human data available in the literature. Plicamycin (Fang et al., 1992; Goodman and Gilman, 1996; Ryan, 1977; Solov'ev and Egorenko, 1976), Auranofin (Capparelli et al., 2017), Panobinostat (Novartis, 2016; Van Veggel et al., 2018), Bortezomib (Tan et al., 2019), Lestaurtinib (Hexner et al., 2015), Ponatinib (Narasimhan et al., 2013), Midostaurin (Cooper et al., 2015), Bardoxolone methyl (Hong et al., 2012), TG02 (Pasha et al., 2012), Sapanisertib (Moore et al., 2018), GDC-0084 (Morrissey et al., 2016; Wen et al., 2016), Bimiralisib (Beaufils et al., 2017; Wicki et al., 2018), PF-04691502 (Britten et al., 2014), CUDC-907 (Bao et al., 2011) , Latrunculin B (Atzberger, 2018; Rasmussen et al., 2014), Quisinostat (Venugopal et al., 2013). TDLo: minimal toxic dose, LD50: lethal dose 50%, IP: intraperitoneal, SC: subcutaneous, IV: intravenous, MTD: maximum tolerated dose. CC_50_ obtained from 72-hour treatment with priority compounds on the human cell lines HepG2 and HEK293. Data available at the ReFRAME database (reframed.org). Multimedia component 1
